# Current Perspectives in Giant Cell Arteritis: Can We Better Connect Pathogenesis and Treatment?

**DOI:** 10.3390/medicina60030400

**Published:** 2024-02-26

**Authors:** Daniela Opriș-Belinski, Claudia Oana Cobilinschi, Ioana Săulescu

**Affiliations:** 1 Department of Rheumatology and Internal Medicine, Carol Davila University of Medicine and Pharmacy, 050474 Bucharest, Romania; danaopris0103@yahoo.com (D.O.-B.); ioana_oprisan@yahoo.com (I.S.); 2 Department of Rheumatology and Internal Medicine, Sfânta Maria Clinical Hospital Bucharest, 011172 Bucharest, Romania

**Keywords:** giant cell arteritis, large-vessel vasculitis, pathogenic pathways, glucocorticoids, novel drugs

## Abstract

Giant cell arteritis (GCA) is a large-vessel vasculitis affecting elderly patients and targeting the aorta and its main branches, leading to cranial and extracranial manifestations. The mechanism behind the ischemia is a granulomatous-type inflammation with potentially critical lesions, including visual loss involving the ophthalmic artery. Despite significant progress in unraveling the pathophysiology of this disease, treatment options still rely on glucocorticoids (GCs) to overcome active vascular lesions and disease flares. However, uncertainty still revolves around the optimal dose and tapering rhythm. Few corticosteroid-sparing agents have proven useful in GCA, namely, methotrexate and tocilizumab, benefiting cumulative GC dose and relapse-free intervals. The future looks promising with regard to using other agents like abatacept and Janus-kinase inhibitors or blocking the granulocyte–macrophage colony-stimulating factor receptor.

## 1. Introduction

Giant cell arteritis (GCA), also known as temporal or Horton’s arteritis after the researcher who initially described it in 1932, is the most frequent idiopathic inflammatory vasculopathy and occurs mostly after the age of 50. Due to a rather large variability in the method of diagnosis (clinical, positive temporal artery biopsy) and/or the 1990 American College of Rheumatology classification criteria [1], as well as possibly some geographical differences, the epidemiological data are quite varied. The estimated annual incidence ranges between 1.1 and 43.6 cases per 100,000 people [2,3], with the highest incidence in the Caucasian population of Northern European descent and North and South Americans, followed by the rest of Europe and Oceania. Also, recently, attention has been drawn to the increase in cases among people of color [4].

It is classified according to the 2012 Revised International Chapel Hill Consensus Conference as a large-vessel vasculitis [5] that especially involves the thoracic aorta and its branches. In the field of rheumatology, GCA remains a medical emergency because the patients carry a high risk for visual loss or stroke. Nevertheless, the more silent aortic involvement has the potential for life-threatening complications (aortic aneurism and dissection) that we all should be aware of [6,7,8,9]. Because long-term medium- and high-dose glucocorticoids are still the mainstay of therapy, disease is also associated with important treatment-related morbidity. Considering the age at diagnosis and the need for prolonged steroid therapy, new insights into the pathogenesis of GCA could offer an important clue to better management and prognosis.

## 2. Etiology and Pathogenesis

GCA is an immune-mediated disease, and, as is the case with most such diseases, a lot of questions are still waiting for an answer [10]. It is characterized by inflammation of the artery wall with a granulomatous pattern, followed by significant loss of vascular smooth muscle cells (VSMC), remodeling of the walls with intimal hyperplasia, and vascular occlusion [11]. From this point of view, GCA is the perfect example of how the role of arteries in the human body is even more essential than a simple route of nutrient delivery or blood pressure regulation, being actively implicated in the body’s defense system. Its etiology and pathogenesis are still a matter of debate, but important advances have been made in recent years that have led to a better understanding of the different mechanisms implicated in the initiation and propagation of specific vascular inflammation.

Although no single instigator of the inflammatory process in the artery wall has been found, epidemiologic studies have helped us detect important predisposing factors, such as female gender, Northern European ancestry, and, the most important one, older age [12,13]. The female predominance in GCA has been found in a multitude of different cohorts, with a female-to-male ratio ranging from 2 to 4, with this gender difference being more obvious in the northern part of Europe [14,15]. The protective role of estrogen on the vessel wall is well known, with early menopause being more frequent in women with GCA [15].

The probability of a genetic influence on the occurrence of GCA is suggested by the geographic variations in incidence rates and by some reports of cases among first-degree relatives [13,14]. As with many autoimmune diseases, GCA susceptibility is related to specific human leukocyte antigen (HLA) class II, mainly HLA-DRB1*0401 and HLA-DRB1*0404 [8,16]. These abnormal amino acids are situated in the antigen-binding region of the HLA molecule, suggesting that GCA might be an antigen-driven disease [17]. More and more data suggest that susceptibility to autoimmune disorders appears after the interaction of multiple genetic factors that regulate the threshold of autoreactivity, and GCA is no exception, being a polygenic disease [18,19]. Non-HLA genetic loci have been associated with this vasculitis, with variants in genes related to angiogenesis or arterial biology, but it is also linked to the innate and adaptative immune response described in this entity (plasminogen, prolyl-4-hydroxylase subunit alpha 2, interleukin-17A, and tyrosine phosphatase non-receptor type 22) [20]. GCA is clearly age related because almost no cases younger than 50 years have been described, and the probability of disease occurrence increases dramatically with age. Immunosenescence and/or artery wall changes are both believed to be implicated. Although there is clear evidence of decreased antibody production and a shortened duration of protective immunity after immunization, age-related immune dysfunction is not yet completely understood [21,22]. It has been demonstrated that elderly people have an imbalance of T cells, with an abnormal response of CD8^+^ CD28⁻Lf T that will trigger the production of an increased amount of interferon γ (IFNγ) [23]. IFNγ will further amplify the T helper 1 (Th1) expression, an abnormal response that has been found to be present in GCA patients. With aging, arterial degeneration is mainly related to atherosclerosis, but this cannot be related to GCA because atherosclerosis is rarely found in temporal arteries, the main site of inflammation in this vasculitis. Instead, a less debated abnormality, calcification of the internal elastic membrane (IEM), has the same vascular distribution, female predominance, and relationship with age as GCA [24]. Foreign-body giant cells associated with inflammatory processes from GCA attack the calcification of IEM, as has been shown by microscopy. The reason only a minority of the population develops vasculitis might be related to genetics or perhaps a particular response to infections. The higher infection rate in GCA patients compared with age-matched controls, especially in the upper respiratory tract [25], and the seasonal variations reported by some studies [26] could suggest an environment–infection relationship. Three relatively recent studies, one North American [26] and two Northern European [27,28], that evaluated the timing of diagnosis of biopsy-confirmed GCA reached similar conclusions, namely, a higher rate of occurrence during the summer months, while data from Australasia [29] did not confirm this aspect. It was speculated that some viral (varicella–zoster and parainfluenza virus type 1) or bacterial (Chlamydia pneumoniae and Mycoplasma pneumoniae) microorganisms might be related to GCA etiology, but no proof was found in specimen biopsy [30,31,32,33]. Nevertheless, despite all the data, at this moment, there is still no evidence to support infection as a causal process in GCA, so antivirals or antimicrobials are not recommended for the treatment of these patients.

## 3. Pathogenic Mechanisms

Because inflammatory changes have been found in the arteries of selected vascular beds, it could be speculated that the disease is associated with specific tissue tropism and that factors originating from the wall are implicated in GCA [23]. It is postulated that elements of the artery wall regulate immune responses and may considerably determine the outcome of the inflammatory reaction from these particular sites [22].

In normal conditions, the arterial wall is an immunoprivileged place, with local immune cells maintaining a tolerogenic state [34]. In GCA, this tolerogenic state is lost, and an inflammatory response will start and propagate with the appearance of granulomatous infiltrates, neovascularization, intimal hyperplasia, and occlusion [10]. The three stages of evolution represent the implications of innate immune cells from the artery wall, followed by the appearance of adaptative immune cells with an increased number of lymphocyte T CD4^+^ and, at the end, structural changes of the arteries [11].

### 3.1. Innate Immune System in GCA

The central cellular player of innate immunity in GCA is represented by dendritic cells (DCs). Normal arteries have DCs in the adventitia, right next to the media layer. They have a discontinuous pattern of distribution along the artery and form a complete circle around the circumference of the vessel. These cells act as wall sentinels with a high threshold for tolerogenic capacity [35,36]. DCs offer immunosurveillance functions by detecting pathogen-derived molecular patterns (PAMPs) through toll-like receptors (TLRs). They respond to bacterial products like bacterial lipopolysaccharide but also to tissue damage, sensing endogenous ligands like fibronectin [37,38]. DCs from the artery wall are in a normal condition in an immature state, conferring equilibrium in the tolerance state. When this equilibrium is gone, mostly under the effect of different cytokines like granulocyte–macrophage colony-stimulating factor (GM-CSF), DCs will express a very active phenotype, promoting activation of T cells via a milieu of cytokines and chemokines that will be released. It is now clear that vascular DCs make a major contribution to initiating and sustaining mural inflammation. Interestingly, a paradigm appears in relation to the aging and tolerogenicity of DCs. It is well known that myeloid and plasmacytoid DCs are less responsive to TLR stimulation in the elderly, suggesting a less inflammatory state, but aging DCs will also exhibit fewer anti-inflammatory functions [39]. Aged DCs are not effective in priming and recruiting T cells or maintaining tolerance to themselves. They become active immune DCs, enabling an inflammatory state and subsequent tissue damage [10]. Vascular DCs offer clues to tissue tropism in GCA. GCA selectively targets the extracranial branches of the aorta, with a predilection toward arteries from the head, neck, and upper extremities [11]. Moreover, skip lesions that are found in arterial biopsy reflect the arrangement of DCs in the walls. Studies conducted on severe combined immunodeficient mice showed that each artery has a specific immune function that is related to a specific combination of TLR acting as a fingerprint, and this might be the clue for future treatments [13].

As part of the innate immune system, macrophages are typically not found in healthy arteries. The pathognomonic hallmark of an arterial lesion in GCA is a multinucleated giant cell from the granuloma, demonstrating the importance of these cells in the pathogeny of GCA [13]. Macrophages are recruited by activated DCs and will differentiate into M1 and M2 phenotypes, showing different functional profiles according to phenotype and positioning in the adventitia, media, or intima. In the adventitia, M1 macrophages are specialized in the production of the proinflammatory cytokines interleukin (IL)-6 and -1 as well as tumor necrosis factor alfa (TNF-α). In the media, M1 macrophages secrete reactive oxygen intermediates and matrix metalloproteinases, damaging the artery wall by destroying endothelial cells and vascular smooth muscle cells. M2 macrophages are situated between intima and media, releasing proangiogenic growth factors like vascular endothelial growth factor (VEGF), fibroblast growth factor (FGF), and platelet-derived growth factor (PDGF), explaining the thickening of the arterial wall and vessel occlusion [40]. It has been postulated that macrophages and DCs both elicit the same effect on the T-cell inflammatory response. Studies using bioengineered human arteries have investigated this theory. Activation of these cells via the TLR-4 pathway demonstrates that only DCs can recruit and activate T cells, while macrophages alone are not capable of promoting T-cell activation [41].

### 3.2. Adaptative Immune System

It is well known now that the innate immune system represents the start of the pathogenic process in GCA-recruiting cells in the artery wall, but the effect of tissue-damaging inflammation is part of the adaptative immune response. Normal arteries are free from T-cell infiltration. They are recruited mainly by DCs, change into effector cell types, and infiltrate the wall. CD4^+^ T cells are necessary for intensifying and maintaining inflammatory changes in the artery wall. Depletion of T cells in mice implanted with GCA temporal arteries stops disease progression and inhibits macrophage activity, making the granulomatous lesion disappear, suggesting that both DCs and T cells have a key role in GCA lesions [42,43].

In GCA, two CD4^+^ T cells have been described: type 1 helper T cells (Th1) and type 17 helper T cells (Th17). They need signals from different antigen-presenting cells. Th1 cells are stimulated by IL-12 and IL-18, while Th17 cells require IL-1β, IL-6, IL-21, and IL-23 [44]. In untreated patients with active disease, Th1 and Th17 cells are found not only in the arterial wall but also in the circulation, associated with high expression of the effector cytokines interferon gamma (IFN-γ) for Th1 and IL-22 and IL-17 for Th17 [16]. The two different subtypes of T cells, Th1 and Th17, have different susceptibilities to respond to glucocorticoids, with only Th17 being highly sensitive to treatment. These might explain the rapid decline in inflammatory reactants when treatment with glucocorticoids is started, but the persistence of Th1 cells and IFN-γ despite treatment might be responsible for the chronic, relapsing evolution of GCA [10].

In addition to effector T cells, regulatory T cells (Treg) are also important. In normal conditions, they have an important role in preventing autoimmunity by maintaining a normal immune tolerance. Under the influence of IL-6, in GCA patients, Tregs have a dysfunctional pattern and contribute to the production of the proinflammatory cytokine IL-17 [44,45,46]. Anti-IL-6 treatment contributes to the restoration of the Treg immune response, blocking the vicious circle of proinflammatory cytokine overproduction.

The implications of T cells in the pathogenesis of large-vessel vasculitis also come from data accumulated after the use of checkpoint inhibitors in cancer. Drug-induced GCA appeared after the use of ipilimumab, an immunomodulator that inhibits the checkpoint molecule cytotoxic T-lymphocyte-associated protein 4 (CTLA-4) [45,46]. Excessive accumulation of activated T cells into affected temporal arteries has also been linked to malfunction of another checkpoint: programmed death 1 (PD-1)/programmed death ligand 1 (PD-L1) (32). All these data confirm that immune checkpoints—necessary to prevent excessive accumulation, retention, and activation of T cells—are deficient in GCA [47].

Lesional CD4^+^ T cells are polyfunctional. Different types of cytokines have been tracked in the GCA wall, including IFN-γ, IL-2, IL-17, IL-21, IL-22, and GM-CSF. The dominant cytokine is IFN-γ, a high amount of which is found in the vascular wall but also in the periphery. It is a major effector cytokine, being responsible for the activation of macrophages, DCs, and endothelial cells. IFN-γ^+^ T cells are dependent on Janus kinase and signal transducer and activator of transcription (STAT) action, with a low response to glucocorticoids; therefore, blocking the JAK pathway could probably be a better solution [36]. IL-22 might represent the link between immune cells and stromal cells, while GM-CSF is an important activator of macrophages and links these cells by their granuloma appearance. IL-17 is a proinflammatory cytokine that is easily suppressed by therapy with glucocorticoids and has a pleiotropic effect on a large number of cells: macrophages, endothelial cells, vascular smooth muscle cells (VSMC), and fibroblasts. IL-17 is linked to IL-6 because Th17 differentiation of T cells and malfunction of Treg are regulated by IL-6 [43,46].

It is evident now that, from the adaptative immune response point of view, GCA is a T-cell-dependent disease, although B lymphocytes are also present in GCA lesions, mainly forming tertiary lymphoid structures. They have an important role in activation of T cells via secretion of proinflammatory cytokines, but the main role of B cells—antibody production—remains unsignificant in GCA [46].

### 3.3. Vascular Inflammation, Remodeling, and Occlusion

After the immune activation in GCA, an intensifying loop follows, culminating in full transmural inflammation, vascular wall lesions, and remodeling. Macrophages are very important in this process, with both phenotypes—proinflammatory (M1) and reparative (M2)—acting together. They contribute to the local and systemic expression of the proinflammatory cytokines IL-1β, TNF-α, and IL-6, associated with local symptoms and elevated inflammatory markers. Angiogenic factors (VEGF, FGF, and PDGF) promote the appearance of new vessels in vascular lesions of GCA, amplifying vascular inflammation through new leucocytes that invade the vessel wall in continuum cascades. Angiogenesis seems to have a dual role in GCA as an important part of chronic inflammation that can still compensate for ischemia at distal sites [30].

Generally, chronic inflammation is followed by damage, and GCA is not an exception. Depletion of VSMC appears secondary to the presence of cytotoxic lymphocytes and activated macrophages. GCA lesions have a proteolytic predominance state, with an increase in metalloproteinase, disrupting elastic fibers, and starting vascular remodeling [16,22]. Growth factors like PDGF or endotelin-1 are released by macrophages and damaged VSMCs. They favor myofibroblast mobilization, migration, and proliferation with intimal hyperplasia. These vascular remodeling factors have a poor response to glucocorticoids, suggesting that specific therapeutic approaches are needed in GCA in order to positively impact vascular remodeling and stenosis [42].

**Figure 1:**
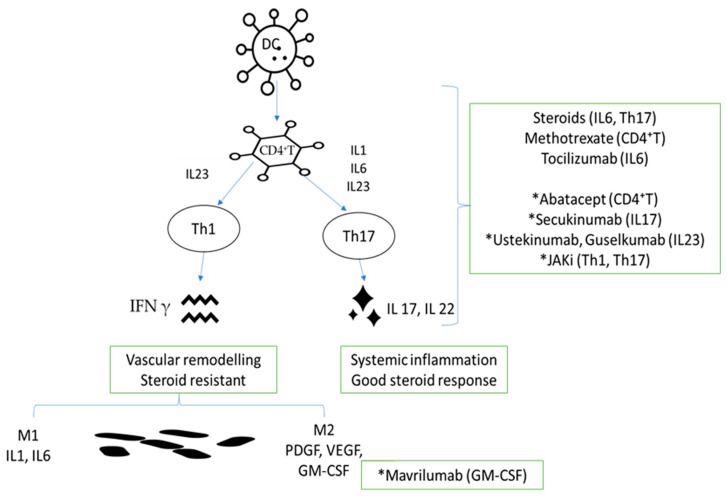
Link between pathogenic mechanisms and already approved or investigational molecules in GCA. DC, dendritic cell; CD4^+^ T, lymphocyte T CD4^+^; IL-23, interleukin-23; IL-1, interleukin-1; IL-6, interleukin-6; IL-23, interleukin-23; Th1, type 1 helper T cells; Th17, type 17 helper T cells; IFN-γ, interferon γ; IL-17, interleukin-17; IL-22, interleukin-22; M1, macrophage M1; M2, macrophage M2; PDGF, platelet-derived growth factor; VEGF, vascular smooth muscle cells; GM-CSF, granulocyte–macrophage colony-stimulating factor; * investigational product.

A better understanding of pathogenic mechanisms will increase the possibility of a more efficient approach with fewer disease or treatment complications. From the pathogenic point of view, Figure 1 shows possible targets that are already-approved or under investigation in GCA treatment.

## 4. Treatment Options in GCA

In the presence of high suspicion or confirmed GCA, treatment should be promptly initiated, which represents a key point in patients’ future disease outcomes. Current available literature provides consistent data on management from American (American College of Rheumatology, ACR), European (European League Against Rheumatism, EULAR), and British (British Society for Rheumatology, BSR) guidelines, but multiple trials are still in search of novel therapies.

As in other immune-mediated vasculitis, the treatment of GCA initially aims for a remission induction phase, followed by the maintenance of a stable disease course. According to GCA-related organ involvement or patient comorbidities, management should be tailored to obtain disease control and reduce the risk of future flares.

### 4.1. Where do We Stand in Treating GCA? What Worked and What Failed? Current Treatment Recommendations

Glucocorticoids (GC) are the mainstay of GCA treatment for every new-onset patient. Their role in rapidly reducing inflammation through both genomic and non-genomic mechanisms helps limit subsequent vascular damage. The preferred mode of administration is still a matter of debate in view of the lack of clear results from randomized control trials (RCTs). The 2018 update of EULAR guidelines [48] recommends starting daily administration of 40–60 mg of prednisone equivalent, then tapering within 2–3 months to 15–20 mg daily, and, after 1 year, to ≤5 mg daily. The treatment should be administered once a day. Other guidelines, like the one of the British Society for Rheumatology (BSR) on the diagnosis and treatment of GCA [49], align with the same recommendation to initiate high-dose GC over moderate- or low-dose GC and for a daily regime to be selected over an alternate day in order to prevent relapses. One slight difference between the two guidelines is found when tapering, with the British one suggesting continuing at the same initial dose until GCA symptoms and acute-phase markers resolve. If clinical remission is achieved and the prednisolone dose is more than 20 mg/day, it should be decreased by 10 mg every 2 weeks. Meanwhile, if in remission, the daily dose of prednisolone between 10 and 20 mg/day should be reduced by 2.5 mg every 2 to 4 weeks. The same regimen was used in the control arm of the GiACTA trial [50]. Response to treatment in terms of constitutional symptoms or headache is usually rapidly observed in the first 24–48 h after GC initiation. The lack of symptom alleviation should prompt reconsideration of the diagnosis of GCA, especially if temporal artery biopsy or ultrasound findings are inconsistent [48].

On the other hand, GC are well known to induce a series of adverse events like infections, hypertension, diabetes mellitus, osteoporosis and fractures, gastrointestinal bleeding, glaucoma, and cataracts [51], which should be taken into account, especially in frail patients. One of the earliest and most cited observational studies that evaluated the rate of glucocorticoid-related adverse events [52] in GCA patients identified that 86% of patients had at least one adverse event, while two or more events occurred in 58% of patients, with the most frequent ones being cataracts, fragility fractures, and infections. Advancing age and high cumulative dose were found to be the main risk factors, and this aspect therefore represents an important problem taking into account the particularities of the patient population.

According to EULAR recommendations [48] in patients who experience GC intolerance or for whom there are considerable threats to long-term treatment, adding a biological agent like tocilizumab (TCZ), a humanized monoclonal antibody against the interleukin-6 receptor, might be of use. Meanwhile, because it can facilitate GC dose reduction and long-term use, the more recent ACR guideline [53] recommends adding TCZ to every newly diagnosed GCA if it is available and cost-accessible. However, it could be accompanied by an increased infection rate, transient dyslipidemia, or a potential risk of intestinal perforation, as proven in patients with rheumatoid arthritis [54]. Moreover, by drastically decreasing the synthesis of C-reactive protein (CRP), an essential follow-up inflammatory marker, the reliability of disease monitoring becomes questionable. The initial studies on TCZ in GCA that led to actual recommendations were published in 2017 and 2019, respectively [50,55]. In a one-year clinical trial, Stone et al. included 251 patients and used subcutaneous TCZ to assess the GC-sparing effect at 52 weeks and disease remission. The authors noted that weekly or biweekly TCZ with a 26-week GC taper proved superior to a 26- or 52-week GC taper and placebo in obtaining a consistent GC-free remission. The 2-year extension of this trial confirmed that almost half of the patients maintained the GC-free period while on TCZ, although disease reactivation still occurred in patients interrupting biological treatment. This study confirmed that the cumulative GC dose was lower in GCA patients who started on TCZ and that there were no new safety issues during the follow-up period [55]. A recently published review using Cochrane selection methodology evaluated two RCTs and included 281 GCA patients. The common results showed that TCZ was superior to placebo in terms of patients with sustained remission, time to relapse, and the use of escape therapy, defined as the need to use GC outside the pre-established study protocol. Both RCTs found infection to be the most frequent adverse event. As we move away from the moment of approval, multiple real-world data are beginning to appear that confirm results similar to those in randomized trials [56,57]. Going back to clinical studies, the minimum duration of GC use was 26 weeks. Consequently, in light of these data, the question was raised as to whether TCZ could be used for induction in monotherapy or only with an initial dose of corticosteroids. Two small clinical studies that included 18 patients each, without a control arm and with a relatively similar design, tried to respond to this question. Muratore et al. [58] included active GCA patients that received 500 mg i.v. methylprednisolone for three consecutive days, followed by weekly s.c. tocilizumab until week 52. Activity was assessed by PET/CT. The primary endpoint of a reduction in the PET vascular activity score (PETVAS) was achieved at 24 and 52 weeks. The other study, the GCA treatment with Ultra-Short Glucocorticoids and Tocilizumab (GUSTO) trial [59], used the same scheme of i.v. GC and s.c. TCZ, but in between them, one i.v. infusion of 8 mg/kg was added. The primary endpoint evaluated the proportion of patients who had remission within the first four weeks and showed that only 25% achieved it. Finally, as per the published results, 78% had remission within 24 weeks, and 72% showed no relapses up to 52 weeks. However, due to the small number of patients, the lack of a control arm, and the short duration of the evaluation, it is still too early to draw a conclusion, and cortisone still maintains its importance in the management of GCA.

The American College of Rheumatology (ACR) recommends adding TCZ to every newly diagnosed GCA if it is available and cost-accessible as it can facilitate GC dose reduction and long-term use. In patients who experience extracranial vessel involvement, adding an immunosuppressive treatment per primam seems to be a valid option according to ACR [53].

Apart from biological agents, methotrexate (MTX) can represent a valid option for patients with difficulties with GC dose reduction or who experience adverse events. Despite older study results with a heterogeneous patient population, MTX has been proven to lower the risk of relapse and reduce the total GC dose. Apart from three studies evaluating MTX over placebo in GCA but using a lower dose (10–15 mg oral administration) and with a short follow-up of approximately two years, a meta-analysis indicated a reduction in second relapse risk in half of the patients (51%). In this publication, Mahr et al. extracted data from 161 patients (84 MTX and 77 placebo) and found that there was a significant decrease in the cumulative GC dose at 48 weeks, and patients receiving MTX were more likely to discontinue GC up until 24 weeks [60]. In 2019, Koster et al. retrospectively evaluated 83 GCA patients for a median of 4 years and divided them into patients on GC monotherapy or receiving GC and MTX [61]. Patients in the latter group showed a significant reduction in disease relapse rates when compared to the GC-alone group, namely, from 11.8/10 person per year to 3.72/10 person per year. However, no GC-sparing effect was proven in this study. Moreover, patients who started on MTX had higher total GC doses from the interval prior to immunosuppression [61]. No head-to-head comparison between MTX and TCZ has yet been performed; thus, the superiority of agents is difficult to prove. However, results from a recruiting study on efficacy and costs (NCT03892785, METOgiA) will be available for interpretation in the future.

Despite a considerable proportion of patients who do not relapse under GC monotherapy with equivalent of prednisolone under 5 mg/day, a dose that is tolerated by EULAR, after one year, there are still patients who experience disease flares. Thus, in minor relapses, an increase in GC to the last effective dose might be sufficient to bring the patient back into remission. On the other hand, in confirmed major relapses with potential organ damage from vessel inflammation, the GC dose should be re-escalated up to 40–60 mg prednisone equivalent, as in new-onset disease. Additionally, in patients who experience relapse, a GC-sparing agent (TCZ or MTX) should be initiated, or if the subject is already on treatment, the agent should be switched or dose-adjusted for better long-term control of the disease. There is scarce data regarding therapeutic options in cases of disease re-activation, especially since MTX studies only included newly diagnosed GCA patients. However, the previously mentioned meta-analysis on the role of MTX in GCA proved a reduced risk of both first and second relapse in patients receiving MTX, indicating that the immunosuppressant can be of use in patients who exhibit disease reactivation. The GiACTA trial that showed the benefit of TCZ on GC-free remission enrolled both new and relapsing GCA patients, suggesting the benefit of the anti-interleukin-6 in this patient population [50]. One large Spanish multicenter study involving 471 patients found similar effectiveness and safety in patients with newly diagnosed GCA when compared to those with refractory/recurrent GCA [62]. The ACR recommends the addition of a nonglucocorticoid immunosuppressive agent in cases of relapse but mentions the superiority of TCZ in patients with cranial symptoms. Moreover, the American guidelines mention the use of intravenous (i.v.) TCZ as an alternative [53].

Current available therapeutic options in GCA and originating clinical studies are summarized in Table 1. 

Other possible adjunctive immunosuppressive therapies (see Table 2), either synthetic (leflunomide, azathioprine, cyclophosphamide, and dapsone) [63,64] or biologic (TNF-α blockers and abatacept), are not included in current recommendations due to low-quality or negative results (cyclosporine) [65]. Due to the similarities with methotrexate, which until the approval of tocilizumab was considered the main steroid-sparing agent, leflunomide was among the most evaluated drugs in several observational studies [66,67,68]. However, although the results were not negative, more data are needed. TNF-α blockers were among the first biologics studied, but they showed negative results. In a double-blind, multicenter, controlled trial in patients with newly diagnosed GCA, at a 6-month final evaluation, the addition of 10-week treatment of adalimumab 40 mg every other week to prednisone did not increase the number of patients in remission [69]. One multicenter randomized control trial of 5 mg/kg infliximab [70] failed to prove relapse-free efficacy at 22 weeks in GCA patients with glucocorticoid-induced remission, so the study was stopped earlier than the initial 54 planned weeks. Rituximab, an anti-CD20, showed benefit for refractory GCA according to two case reports [71,72]. Abatacept, a CTLA-4 agonist that selectively modulates co-stimulation, was evaluated in a multicenter trial that included 49 GCA patients and showed benefit for a longer relapse-free interval compared to placebo and a lower GC exposure [73]. Abatacept is also mentioned in ACR guidelines as a useful steroid-sparing agent [53].

### 4.2. Adjacent Therapeutic Options

Given that GCA affects elderly patients who are already prone to a higher risk of cardio- or cerebrovascular events [76], additional therapies have been discussed. Up to present, low-dose aspirin is not routinely recommended because it has not been proven useful for prophylaxis. One quite old observational study [77] suggests that low-dose aspirin decreases the rate of visual loss and cerebrovascular accidents, but this has not been proved by large randomized clinical trials. ACR conditionally recommends aspirin in patients with severe vertebral or carotid involvement with impaired blood flow who would benefit from aspirin in order to reduce the occurrence of ischemic events. However, if the patient is already on antiplatelet therapy or anticoagulants for a specific indication, treatment should not be interrupted [53].

Despite the fact that some observational studies [78] have suggested a certain protective effect of statins in GCA-related vascular inflammation, the evidence is not sufficiently strong to recommend them routinely in these patients. Nevertheless, due to patient characteristics (older than 50 years) and cortisone treatment, cardiovascular risk assessment should be evaluated in all individuals and managed according to current guidelines. A French population-based study [79] concluded that among the 103 GCA patients followed, statin use was associated with reduced cardiovascular hospitalizations.

### 4.3. Emergency Management in GCA

Visual loss represents a major risk in GCA, either at disease onset or during flares, and it is mostly irreversible. EULAR states that intravenous methylprednisolone can be used (0.25–1 g) for up to three days. However, if the intravenous route is unavailable, high-dose oral GC should not be delayed [48]. In patients with other signs of GCA-related cranial ischemia, namely, amaurosis fugax or stroke, according to ACR [53], intravenous GC pulse therapy can be administered only after evaluating patient risks like age, comorbidities, and preferences.

In 2021, a study aimed to assess the prevalence of visual impairment in a GCA cohort with 186 patients receiving TCZ. At enrollment, visual involvement was detected in 38% of patients and visual loss in 11%. Two patients experienced visual loss through acute ischemic optic neuropathy (AION) while on TCZ, and despite receiving prompt GC pulse therapy, there was no eye recovery. In addition, it appeared that the vessel structure was severely impaired, possibly from both GCA and atherosclerosis, which could not be reversed despite intensive treatment [80].

### 4.4. Treat-to-Target Approach

The treat-to-target approach is already the standard of care for a multitude of pathologic conditions, including immune-mediated rheumatologic disorders like rheumatoid arthritis [81], spondyloarthritis [82], and systemic lupus erythematosus [83]. The main goal is to provide guidelines to achieve and maintain remission in order to limit or postpone the accumulation of irreversible damage.

As GCA is a disease found mainly in a population that has already accumulated different comorbidities, like vascular disease, osteoporosis, and diabetes, treatment should be tailored to control disease activity while limiting complications or worsening of already present diseases. Released for the first time in January 2024, the treat-to-target recommendation in GCA [84] offers tremendous support for clinicians and reinforces the need for urgent treatment, which is possible with a multidisciplinary approach in order to avoid ischemic complications. The first of six recommendations stipulates that treatment should aim for remission, defined as “the absence of clinical symptoms and systemic inflammation”, and it is closely linked to the second recommendation that treatment “should also aim to prevent tissue ischemia and vascular damage”. As damage accrual might also be related to treatment, adapting it to disease severity and activity but also to comorbidities will limit the accumulation of irreversible lesions (3rd and 4th recommendations). The maintenance phase should use the lowest effective dose of medication, and patients should be evaluated periodically for 1 to 4 weeks until remission is reached and, thereafter, when stable on therapy, between 3 and 6 months (5th and 6th recommendations). A correct differential diagnosis at every visit will avoid unnecessary escalation of medication because headache as well as periarticular or neurologic disease might also contribute to the clinical complaints [84]. The hope is that, like other diseases, the treat-to-target approach in GCA will contribute to a better quality of life for patients, helping physicians to make better decisions concerning patients’ treatment.

### 4.5. What does the Future Hold in Terms of GCA Treatment?

As one of the most frequent systemic vasculitis with a marked potential for morbidity and mortality, GCA arouses an increasing interest in identifying effective therapeutic solutions, especially as there are still multiple unmet needs. For the moment, multiple promising molecules are in phase II and III studies (Table 3).

The effect of Janus kinase (JAK) inhibitors, namely, tofacitinib (JAK1 and JAK3 inhibitors) and baricitinib (JAK1 and JAK2 inhibitors) [16] has been assessed in small pilot studies (less than 20 patients), and they seem to be well tolerated, but larger cohort trials are needed to evaluate the GC-sparing potential of this drug class [85]. Upadacitinib (a second-generation selective JAK1 inhibitor) is currently under evaluation in a phase III trial called SELECT-GCA, which plans to enroll more than 400 patients with active GCA [86].

The potential role of IL-17 inhibition was tested using secukinumab 300 mg s.c., an IL-17A blocker. Published data from the 52-week phase II randomized clinical trial TitAIN showed promising results in association with the 26-week prednisolone taper regimen [75], meaning a higher sustained remission rate along with a longer time to the first GCA flare versus placebo. The phase III study is now recruiting (NCT 04930094).

Blocking the effect of the granulocyte–macrophage colony-stimulating factor (GM-CSF) receptor with mavrilimumab showed in vitro benefits by reducing the T-cell activation process, the CD34 cells, and the neoangiogenic phenomenon in GCA [87]. The phase II clinical trial [74] included patients in glucocorticoid-induced remission who were randomized (3:2 ratio) to mavrilimumab or placebo. The primary efficacy endpoint of time-to-first adjudicated GCA flare by week 26 in all treated patients was statistically significant.

One retrospective study analysis of GCA patients from the French Study Group for Large-Vessel Vasculitis found positive results in six patients treated with anakinra, an IL-1 receptor antagonist [88]. Currently, a phase II clinical trial called GiAnT (Giant Cell Arteritis and Anakinra Trial) is recruiting (NCT02902731).

### 4.6. Treatment Tailoring and Long-Term Monitoring

Modern medicine empowers physicians to tailor treatments according to patients’ preferences, comorbidities, previous experiences, and disease course. However, in GCA, personalized treatment does not allow such a wide range of agents. Knowing that GCs are the mainstay treatment in both new GCA and relapse and dose mastering can make the difference between active and quiescent disease.

Tapering GC has proven to be a challenge in GCA because the rate of relapse reaches up to 75% once it is reduced. Thus, EULAR recommends reaching a dose of 15–20 mg daily in the first 2–3 months and aiming for less than 5 mg per day after one year. A clinical trial CORTODOSE (NCT04012905) is currently planning on evaluating which GC tapering regime is superior, namely, a short or a long conventional one. Apart from GC, MTX or TCZ seem to be the only viable options at present, and their use comes down to accessibility, costs, and patient history.

## 5. Conclusions

The prognostic features of patients with GCA have changed significantly in recent years, but there is still a lot to be done. Preventing not only disease-related damage but also the side effects of prolonged corticosteroid use represents a challenge for the treatment of the disease. Moving forward with an understanding of the pathogenic mechanism, we will be one step closer to achieving these goals.

## Figures and Tables

**Table 1:** Available therapeutic options in GCA according to current guidelines.

Level of Evidence	Patient Population	Intervention	Clinical Endpoints	Surrogate Endpoints
Randomized, double-blind, placebo-controlled, phase 3 trial (GiACTA trial) Stone J.H, PMID 28745999	Patients > 50 y.o, active GCA within 6 weeks from baseline, history of GCA-related elevated ESR	162 mg sc TCZ weekly or every other week or placebo combined with a 26-week prednisone taper	Rate of sustained GC-free remission at week 52	Cumulative prednisone dose, incidence of the first flare after remission, quality-of-life changes (SF-36), patient’s global assessment of disease activity (VAS)
Multicenter, randomized, double-blind, placebo-controlled, parallel-group study (GiACTA extension) Stone J.H, PMID 38279390	GiACTA patient population	Investigators’ discretion: no treatment, TCZ, GC, MTX, or combinations	Maintenance of efficacy 1 year after discontinuation of TCZ	Effectiveness of retreatment with TCZ after relapse, and the long-term GC-sparing effect of TCZ
RCT review, *Cochrane methodology* Antonio A.A, PMID 34420204	Patients > 50 y.o, with new-onset or relapsing GCA (biopsy-proven/angiography); minimum follow-up of 6 mo	TCZ of any dosage regimen (alone or with GC) *versus* therapy without TCZ	Effectiveness and safety of TCZ ± GC *versus* no TCZ	N/A
Meta-analysis of 3 RCTs Mahr A.D, PMID: 17665429	Patients with newly diagnosed GCA	Initial high dose GC + MTX 7.5 to 15mg/week or placebo	Efficacy and safety of adjunctive low-dose MTX in GCA	GC cumulative dose and discontinuation rates
Retrospective chart review Koster M.J, PMID: 30647171	Patients with GCA (temporal artery biopsy and/or radiographic evidence of LVV) single-institution cohort (1998–2013)	MTX+GC *versus* GC alone	Effect of MTX on relapse risk and GC use in GCA cohort	N/A
Single center, phase 2, randomized, double-blind, placebo-controlled trial Villiger P.M, PMID: 26952547	Patients with newly diagnosed or recurrent GCA	Oral GC and either TCZ at 8 mg/kg or placebo, both intravenously	Efficacy and safety of TCZ (complete remission at week 12)	Relapse-free survival at week 52, time to first relapse after induction of remission, cumulative GC dose
Multicenter, randomized trial Langford C.A, PMID: 28133925	Patients with newly diagnosed or relapsing GCA	ABT 10 mg/kg iv on days 1, 15, 29, week 8 + prednisone 40–60 mg/day; double-blinded randomization at week 12	Efficacy of ABT to placebo in GCA duration of remission (relapse-free survival)	Toxicity of ABT

ABT, abatacept; GCA, giant cell arteritis; GC, glucocorticoids; MTX, methotrexate; RCT, randomized controlled trial; TCZ, tocilizumab; VAS, visual analogue scale; N/A, not applicable; LVV, large-vessel vasculitis.

**Table 2:** Tentative treatments in GCA.

Tentative Treatments in GCA	Study Profile	Significant References	Comments
***Non-Biologic Immunosuppressants*** (azathioprine AZT, leflunomide LFN, mycophenolate mofetil MMF, hydroxychloroquine HCQ, dapsone, and cyclophosphamide CYC)	Small trials/case series Observational studies	[63,64,66,67,68]	- HCQ did not show efficacy; - Cyclosporine A: no GC-sparing role; - CYC efficient in GC-resistant disease but significant adverse events; - LFN lowered rate of GCA relapse and proved GC sparing effect
***TNF Inhibitors*** (infliximab, adalimumab, etanercept)	RCTs, small study size	[69,70]	- IFX: no significant role on relapses or GC cumulative dose; higher risk of infections; - ADL: not beneficial; - ETA: positive results on GC-free remission and GC cumulative dose but small study;
***JAK Inhibitors*** (tofacitinib, baricitinib, upadacitinib)	Phase 2 pilot studies Phase 3 trial	[16]	- Insufficient evidence
***B-Cell Depletion*** (Rituximab)	Case reports		- RTX: efficacy in refractory GCA
***Anti-IL-1β Inhibitors*** (anakinra, gevokizumab)	Case series Multicentre RCT	NCT02902731	- ANK: GCA symptoms, inflammatory markers and imaging improvement - GVZ: early trial termination
***GM-CSF Pathway Inhibitors*** (mavrilimumab)	Phase 2 RCT	[74]	- MAV: efficacy in 83% patients in sustained remission at week 26
***IL-17 Inhibition*** (secukinumab)	Case reports	[75]	- SEK: results on remission maintenance

**Table 3:** Potential Therapeutic Agents for GCA in Ongoing Trials, clinicaltrials.gov.

Study Details	Patient Population	Study Status	Primary Aim	Study Design	Trial Identifier
Abatacept for Treating Adults with GCA and TAK	Patients > 15 y.o, diagnosis of GCA and history of active disease within the past 2 months	Completed	Safety and effectiveness of abatacept in treating GCA and preventing disease relapse	Randomized withdrawal design protocol	NCT00556439
Methotrexate Versus Tocilizumab for Treatment of Giant Cell Arteritis: a Multicenter, Randomized, Controlled Trial (METOGiA)	Patients with confirmed GCA and temporal artery biopsy and/or imaging evidence of LVV and/or active GCA witdapsone, and cyclophosphamide (CYC)hin 6 weeks before randomization	Recruiting	Comparison MTX versus TCZ in terms of relapse prevention and cost, 12 months	Multicenter RCT	NCT03892785
Tocilizumab discontinuation in Giant Cell Arteritis (MAGICA)	Patients with GCA on TCZ prior to randomization	Not yet recruiting	The relapse-free survival in groups (immediate vs. gradual discontinuation), 26 week-follow-up	Interventional, phase 3 trial	NCT06037460
Biologics in Refractory Vasculitis (BIOVAS)	Adults and children > 5 y.o with refractory NAAV	Active, not recruiting	Investigation of three biologics (IFX, RTX, TCZ) and placebos to each, in the treatment of refractory (NAAV)	multi-center, randomized, double-blind, placebo-controlled, design	NCT05168475
Ustekinumab for the Treatment of Relapse of Refractory Giant Cell Arteritis (ULTRA)	Patients with GCA, active disease/relapse on recent treatment or on GC	Recruiting	Efficacy of ustekinumab for GCA relapses	Interventional, phase 2 trial	NCT03711448
Phase III Study of Efficacy and Safety of Secukinumab Versus Placebo, in Combination with Glucocorticoid Taper Regimen, in Patients with Giant Cell Arteritis (GCA)	Patients > 50 y.o with GCA, active disease/relapse	Recruiting	Efficacy of secukinumab in combination with a 26-week prednisone taper regimen compared to placebo	Interventional, multi-center, phase III study	NCT04930094
A Study to Evaluate the Safety and Efficacy of Upadacitinib in Participants with Giant Cell Arteritis (SELECT-GCA)	Patients > 50 y.o with active GCA	Active, not recruiting	Assess the safety and tolerability of UPA in GCA and compare withdrawal vs. continuation in sustained remission	Interventional, phase 3 trial	NCT03725202
Giant Cell Arteritis and Anakinra Trial (GiAnT)	Patients > 50 y.o with active GCA	Recruiting	Evaluate anakinra against placebo in addition to GC in the treatment of GCA.	Interventional, phase 3 trial	NCT02902731

ABT, abatacept; GCA, giant cell arteritis; LVV, large-vessel vasculitis, UPA, upadacitinib.

## Data Availability

Not applicable.
